# Bimodal regulation and precision therapy of neutrophil extracellular traps in liver ischemia-reperfusion injury: recent advances

**DOI:** 10.3389/fimmu.2026.1796360

**Published:** 2026-02-26

**Authors:** Peng An, Yi An, Mengwei Chen, Longlong Wu, Rong Wang

**Affiliations:** 1 The Gastroenterology Department of Shanxi Provincial People’s Hospital, Shanxi Medical University, Taiyuan, China; 2 Department of Laboratory Medicine, Taiyuan Peace Hospital, Taiyuan, China

**Keywords:** bimodal regulation, hepatic ischemia reperfusion injury, neutrophil extracellular traps, precision medicine, programmed cell death

## Abstract

Liver transplantation is the definitive therapy for end-stage liver disease, yet hepatic ischemia–reperfusion injury (HIRI) remains a leading cause of early graft dysfunction. Neutrophil extracellular traps (NETs) can support host defense, but dysregulated NET formation during HIRI amplifies sterile inflammation, endothelial injury, and microvascular thrombosis—making NETs an attractive, pharmacologically tractable target. This review integrates recent evidence that NET responses in HIRI are bimodally regulated and stage dependent, with distinct release programs and molecular signatures across ischemia, early reperfusion, and late repair. We summarize key biochemical control nodes governing NET generation and persistence—oxidant signaling, calcium-dependent chromatin remodeling, protease-oxidase feed-forward loops, and platelet–endothelial crosstalk that promotes intravascular NET deposition. We further discuss how NETs reshape the hepatic immune microenvironment, driving inflammatory amplification, immune suppression, and coupling to regulated cell-death circuits, thereby sustaining tissue injury and impairing graft recovery. Translational implications are highlighted through NET-related biomarkers and intervention strategies spanning NET dismantling, inhibition of NET formation, modulation of upstream priming pathways, and liver-directed delivery, including ex vivo machine perfusion as a precision platform. NETs represent a druggable hub linking inflammation, thrombosis, and cell death in HIRI, but timing and selectivity are crucial to avoid compromising antimicrobial defense. Progress requires standardized NET readouts to define therapeutic windows and mechanism-guided combination regimens that selectively suppress pathogenic NET programs to improve graft preservation and post-transplant outcomes.

## Graphical Abstract

**Figure:**
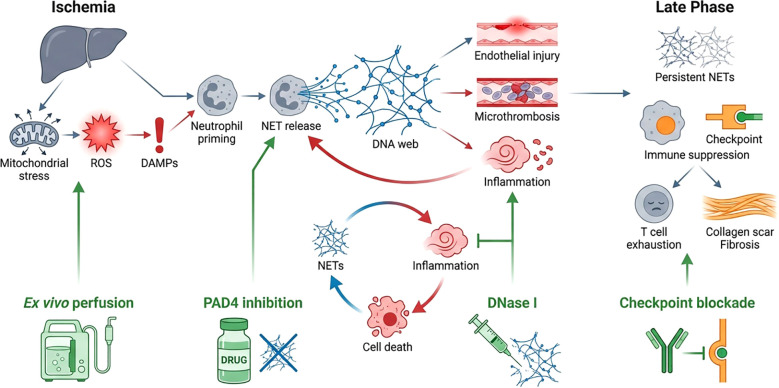

## 1 Introduction

HIRI is a fundamental complication of liver transplantation, in which transient ischemia is followed by a disproportionate inflammatory burst upon reperfusion. Clinically, IRI is tightly linked to early graft dysfunction and failure, reported to occur in a substantial proportion of recipients (1), and it increases the risk of primary nonfunction as well as acute and chronic rejection, thereby remaining a key determinant of transplant outcomes. Mechanistically, reperfusion rapidly triggers redox imbalance and sterile inflammation, leading to hepatocellular death and microcirculatory disturbance. These events are orchestrated by convergent molecular programs—including ROS amplification, Ca²^+^-dependent enzyme activation, damage-associated molecular patterns (DAMPs) signaling through pattern-recognition receptors, and downstream cytokine/chemokine cascades—that recruit and activate innate immune cells. Among these effectors, neutrophils and NETs have emerged as a central molecular hub linking oxidative stress, immunothrombosis, and tissue injury. NET formation is driven by targetable nodes such as NOX2/mitochondrial ROS, PAD4-mediated histone citrullination, neutrophil elastase and myeloperoxidase, and gasdermin-dependent membrane permeabilization, which collectively shape liver IRI progression. Notably, NETs may exert bimodal effects by both aggravating parenchymal damage and modulating host defense and repair, highlighting a rationale for precision strategies that inhibit pathogenic NET pathways while preserving beneficial functions. This review therefore summarizes recent advances in NET biology in liver IRI, emphasizes key actionable molecular targets, and discusses emerging precision therapeutic approaches (2).

## 2 Biological characteristics and regulatory networks of NETs

### 2.1 Molecular basis of NET formation

The mechanism of NET formation involves a multi-level, tightly regulated network. Across these pathways, upstream stimuli are funneled into a limited set of execution events—ROS amplification, Ca²^+^ influx–driven PAD4 activation, chromatin decondensation, and granule protease translocation—ultimately yielding measurable NET readouts such as Cit-H3, MPO–DNA complexes, and extracellular DNA networks (3).At the trigger factor level, studies have shown that superoxide anions derived from mitochondrial complex III can induce NET-related cell death (NETosis) by activating the PKC signaling pathway. This process is closely associated with reactive oxygen/nitrogen species (ROS/RNS) storms (4, 5). Keitaro et al. (6) demonstrated that oxidative damage to mtDNA can synergistically activate the cGAS-STING pathway, further amplifying the ROS/RNS storm effect by enhancing interferon signaling. Additionally, high-mobility group box protein B1 (HMGB1) released by damaged hepatocytes binds to the RAGE receptor, activating the MAPK and NF-κB pathways to promote the release of inflammatory factors (7–9). HMGB1 can also bind to the TLR9 receptor, triggering endoplasmic reticulum stress in neutrophils, leading to the release of the endoplasmic reticulum calcium pool and activation of the calcineurin signaling axis (10–12). Furthermore, Krishnan et al. (13) found that at the core regulatory enzyme level, peptidyl arginine deiminase 4 (PAD4)-catalyzed histone H3 citrullination (H3cit) is a key marker for NET formation, and its specific inhibitor GSK484 significantly inhibits NET release (14, 15).

Additionally, the myeloperoxidase (MPO)-neutrophil elastase (NE) cascade promotes chromatin unwinding by oxidizing the TET2 enzyme (16, 17), forming a self-reinforcing positive feedback loop. The activity of MPO in this system is regulated by phosphorylation of the CDK6-cyclin D3 complex, and blocking this phosphorylation site significantly reduces the extracellular trapping capacity of NETs. These findings provide a molecular foundation for developing spatiotemporally specific regulatory strategies. At the epigenetic regulatory level, the histone H3K27me3 demethylase JMJD3 has been shown to relieve chromatin condensation, with its activity regulated by the ROS-induced JNK signaling pathway (18, 19). Studies on metabolic reprogramming have shown that NETosis is associated with enhanced glycolysis, and PFKFB3-mediated activation of 6-phosphofructokinase-2 supports chromatin unwinding by increasing ATP production (20, 21). The elucidation of these multi-level regulatory mechanisms lays the foundation for developing spatiotemporally specific regulatory strategies. In particular, stage-specific interventions targeting NET formation, such as early inhibition of ROS storms, mid-stage blockade of PAD4 activation, and late-stage disruption of the MPO-NE cascade, have shown significant therapeutic efficacy in sepsis models (22, 23) (**Table 1**).

**Table 1:** Molecular components and signaling pathways involved in NET formation.

Component/pathway	Primary role in NET formation	Upstream activators	Downstream effect	Key experimental readouts	References
NOX2	Generates ROS required for classical NETosis	Microbial ligands, inflammatory mediators, receptor signaling	Promotes NE and MPO translocation and chromatin decondensation	ROS assays, NOX2 inhibition or knockout, NET imaging	(24)
ROS	Central signaling mediator driving chromatin relaxation	NOX2 activation, mitochondrial stress, inflammatory stimuli	Induces protease activation and membrane disruption	ROS probes, ROS scavengers, Cit-H3 and MPO–DNA assays	(25)
PAD4	Citrullinates histones enabling chromatin decondensation	Intracellular calcium signaling	Facilitates chromatin relaxation and DNA extrusion	PAD4 inhibition or knockout, Cit-H3 detection	(26, 27)
NE	Cleaves histones and nuclear structural proteins	ROS-dependent granule permeabilization	Promotes nuclear envelope breakdown and chromatin expansion	NE inhibition or knockout, histone cleavage assays	(28, 29)
MPO	Supports chromatin decondensation and stabilizes NET structures	Granule release following activation	Enhances chromatin unfolding and antimicrobial activity	MPO immunostaining, MPO–DNA ELISA	(30, 31)
GSDMD	Forms membrane pores contributing to lytic NET release	Caspase activation and inflammatory signaling	Facilitates plasma and nuclear membrane permeabilization	GSDMD cleavage assays, knockout models, live-cell imaging	(32, 33)
Calcium signaling	Activates PAD4 and regulates granule mobilization	Receptor-mediated calcium influx	Supports chromatin modification and NET release	Calcium chelation, calcium imaging, PAD4 activity assays	(34, 35)
PKC/MAPK cascades	Transmit receptor-derived activation signals	Protein kinase activation by inflammatory mediators	Promote granule translocation and ROS production	Kinase inhibition, phosphorylation assays, NET quantification	(36, 37)
Autophagy machinery	Facilitates granule trafficking and chromatin processing	Cellular stress and inflammatory cues	Supports chromatin decondensation in select NETosis pathways	ATG protein knockdown, autophagy inhibition, imaging studies	(38, 39)
Mitochondrial ROS and mitochondrial DNA release	Drive rapid NET formation, including mtDNA-rich NETs	Hypoxia-reoxygenation, immune receptor signaling	Initiate mitochondrial DNA extrusion and non-lytic NET formation	mtROS probes, mtDNA quantification, NOX2-independent NET assays	(40, 41)
Pattern recognition receptor signaling	Detects danger signals and amplifies NETosis cascades	DAMPs released during tissue injury	Activates downstream NF-κB and MAPK pathways leading to NET release	Receptor blockade or knockout, cytokine assays, NET imaging	(42–44)
Platelet–neutrophil interactions	Prime neutrophils for localized NET release	Platelet activation during vascular injury	Enhance adhesion, calcium signaling, and ROS-dependent NET formation	Platelet depletion, adhesion blockade, intravital microscopy	(45)

### 2.2 The dual-mode release mechanism of NETs

**Figure 1:**
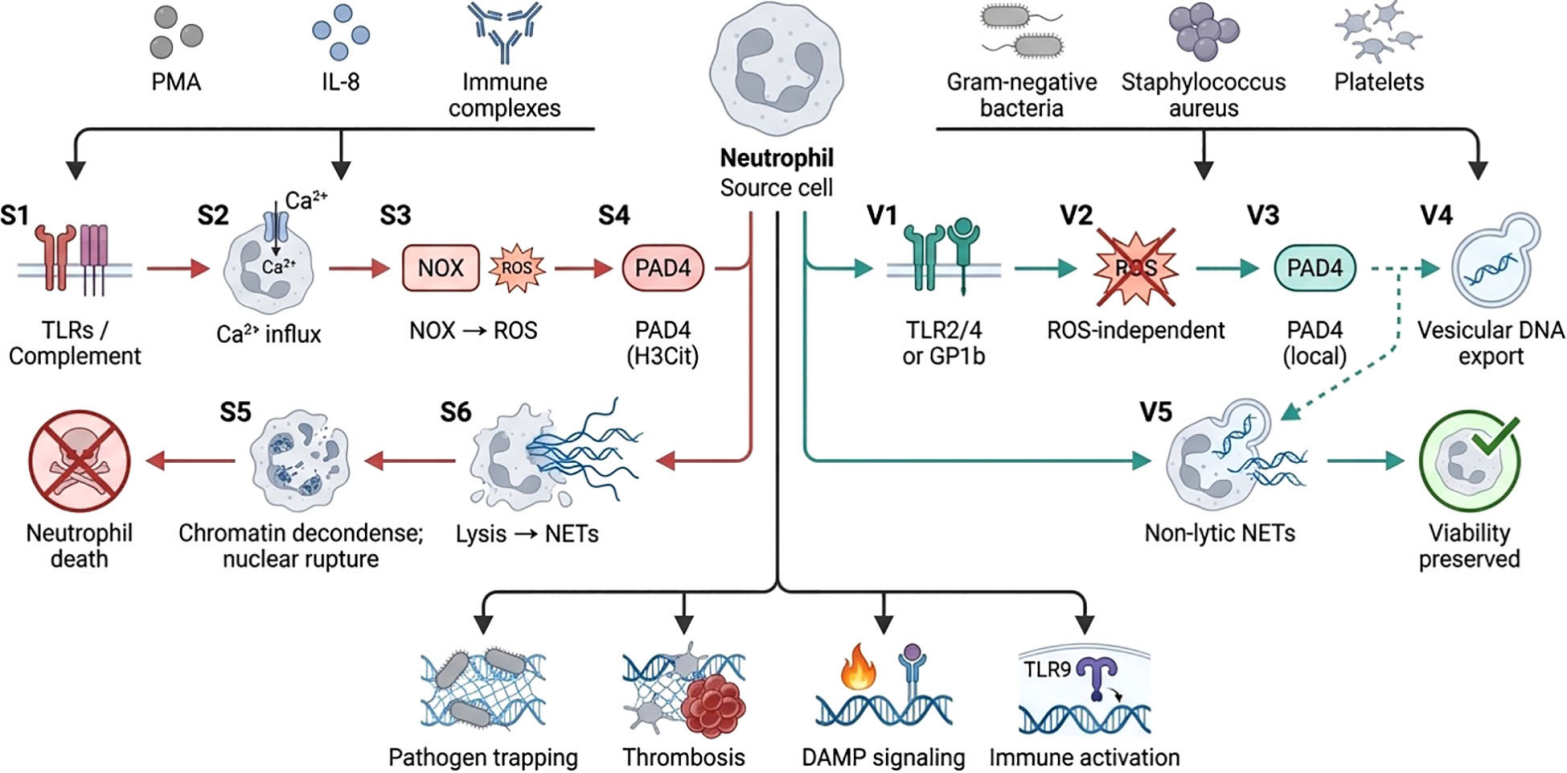
Distinct mechanisms of suicidal and vital NETosis. Suicidal NETosis is initiated by stimuli like PMA, IL-8, or immune complexes activating TLRs and complement receptors, leading to calcium influx and NOX-dependent ROS production that activates PAD4, causing chromatin breakdown, nuclear envelope rupture, NET release through membrane lysis, and cell death. Vital NETosis, triggered by Gram-negative bacteria, Staphylococcus aureus, or platelets via TLR2/4 or GP1b, also activates PAD4 but bypasses ROS, releasing chromatin via vesicles without membrane rupture to preserve neutrophil viability and ongoing immune functions.

The mechanisms underlying NET formation exhibit considerable heterogeneity, with release patterns classified as suicidal NETosis and non-lethal release, based on energy metabolism characteristics and biological effects (**Figure 1**) (46). Importantly, these release modes are associated with distinct downstream signatures: suicidal NETosis typically aligns with robust NOX2-dependent ROS and Cit-H3 enrichment, whereas vital NET release is often characterized by mtDNA-rich extracellular traps and preserved neutrophil viability—features that can be leveraged for time-stratified biomarker interpretation (47). In terms of energy metabolism, suicidal NETosis is largely dependent on the NADPH oxidase complex to generate reactive oxygen species (ROS), which induce chromatin disorganization via PAD4-mediated histone citrullination (48, 49). In contrast, non-lethal release primarily relies on mitochondrial energy, with mitochondrial energy levels decreasing to 70–80% of normal levels, but not being entirely depleted (50, 51). This energy state suggests the presence of a “pause button,” indicating the potential to control or reverse NET release by regulating energy supply, thus highlighting the dynamic regulatory basis of this pathway (52, 53). In terms of genetic material origin, the suicidal pathway is characterized by the release of nuclear DNA, forming a dense network structure (54). Recent studies have demonstrated that non-lethal cellular release primarily involves mtDNA, whose distinct unmethylated CpG motifs exhibit enhanced efficiency in activating the TLR9 signaling pathway (**Figure 1**) (55). Importantly, the pathological significance of the two release patterns contrasts sharply: suicidal NETosis is associated with aggravated hepatocellular injury through the release of proteases and cytotoxic histones, significantly contributing to microthrombosis formation; non-lethal release, on the other hand, forms a reticular structure with DNA fibers that capture pathogens and integrates multiple antimicrobial proteins (e.g., histones and myeloperoxidase) to synergistically kill bacteria, as demonstrated by animal experiments showing a reduction in liver bacterial load (56, 57). The heterogeneous release mechanisms of NETs, along with their precise regulatory networks, not only reveal their functional plasticity in the inflammatory microenvironment but also provide a molecular basis for understanding their dual damage-repair role, as described in subsequent sections (58, 59). This spatiotemporal dynamic biological characteristic enables NETs to exhibit opposing functional spectra in HIRI based on variations in microenvironmental signals (**Figure 1**).

## 3 The dual mechanism of NETs in HIRI

### 3.1 NETs-driven defense-regeneration network: pathogen clearance and homeostasis restoration

NETs exhibit multifaceted regulatory roles in intestinal barrier defense and tissue repair. In pathogen clearance, NETs form a three-dimensional grid structure via their DNA scaffold and guanine tetralyces, capturing Gram-negative bacteria (e.g., *Escherichia coli*) through charge adsorption, thereby reducing bacterial motility (60, 61). Notably, this physical barrier function retains high capture efficiency even under portal hypertension conditions (62), likely due to enhanced matrix adhesion through the interaction between integrin αMβ2 and fibronectin in NETs (63). Additionally, NETs enrich antimicrobial peptides, such as LL-37, through electrostatic interactions. The amphiphilic α-helix structure of LL-37 inserts into bacterial membranes to form ion channels, synergizing with NETs-DNA to significantly enhance bacterial killing efficiency (64). Furthermore, α-defensins released by NETs activate the AMPK/mTOR pathway in intestinal epithelial cells, promoting the autophagic clearance of internalized pathogens (65, 66).

During tissue repair, NETs activate the TLR9/STAT3 pathway in hepatic stellate cells (HSCs) via methylation of CpG motifs, upregulating COL1A1 to promote collagen secretion and drive fibrosis. Concurrently, the released MMP-9 degrades abnormal matrix components and synergizes with VEGF to induce angiogenesis, exhibiting dual functions in matrix remodeling and wound healing during injury repair (67, 68). The S100A8/A9 protein complex within NETs activates the TLR4/MyD88 signaling axis in fibroblasts, promoting IL-10 secretion to suppress excessive inflammatory responses (69, 70). Regarding angiogenesis, VEGF-A encapsulated in NETs activates the FAK/Src signaling axis via integrin αvβ3, inducing endothelial cell pseudopod extension and promoting the formation of a regenerated vascular network (71, 72). NETs-DNA can also activate the STING pathway to induce endothelial cells to secrete ANGPT2, thereby enhancing vascular permeability and angiogenesis (73, 74).

In response to oxidative stress, MPO-catalyzed hypochlorous acid (HOCl) triggers key regulatory events at specific concentrations. HOCl modifies cysteine residues at positions 151/273/288 of the Keap1 protein to form trisulfide bonds, disrupting the Keap1-Nrf2 complex, promoting Nrf2 translocation to the nucleus, and activating antioxidant gene transcription (75–77). Among these, heme oxygenase-1 (HO-1) expression is significantly upregulated, decomposing the pro-oxidative substance heme and reducing lipid peroxidation products such as MDA. NETs also activate the cGAS-STING pathway by releasing mtDNA, inducing the production of type I interferons and further enhancing SOD2 expression to protect mitochondrial function (78–80).

At the non-enzymatic defense level, NETs utilize the unique cation-binding domain of histone H4 to form molecular “protective capsules.” Through electrostatic interactions, these capsules bind to the anionic surfaces of extracellular superoxide dismutase (SOD3), prolonging its half-life and enhancing its targeting to the microenvironment of hepatic sinusoidal endothelial cells, thereby stabilizing mitochondrial membrane potential by clearing superoxide anions (26, 81). Additionally, NETs activate the Wnt/β-catenin pathway in intestinal stem cells via the HMGB1-RAGE axis, promoting the proliferation of Lgr5+ stem cells and epithelial regeneration. NE derived from NETs cleaves the extracellular matrix protein decorin, releasing latent TGF-β1, which drives myofibroblast differentiation through Smad3 phosphorylation (13, 82). This multi-layered defense-repair-homeostasis mechanism highlights the spatiotemporal regulatory characteristics of NETs in intestinal barrier function, with its dynamic balance precisely regulated by PAD4-mediated histone citrullination and the TREM-1/DAP12 signaling pathway (1, 83) (**Table 2**).

**Table 2:** The dual mechanism of NETs in HIRI.

Mechanistic dimension	Protective functions	Pathological consequences	Key molecular mediators	Representative evidence/models	References
Innate immune defense	Enhance pathogen trapping and microbial clearance; maintain early inflammatory containment	Exaggerated neutrophil activation intensifies sterile inflammation and microvascular injury	NE, MPO, ROS, PAD4	Neutrophil depletion, NOX2 or PAD4 knockout, NET quantification in HIRI models	(84, 85)
Tissue protection–regeneration axis	Promote clearance of necrotic debris; recruit reparative immune cells; support early hepatocyte regeneration	Excessive NET burden disrupts hepatocyte membranes, increases permeability, and provokes secondary necrosis	HMGB1 signaling, PRR pathways, extracellular DNA	HMGB1 neutralization, TLR inhibition, antibody-based NET blockade	(3, 86, 87)
Vascular homeostasis	Contribute to temporary hemostasis and containment of endothelial injury	Drive sinusoidal obstruction, platelet aggregation, and microthrombus formation	Platelet–neutrophil adhesion molecules, vWF, histones	Platelet depletion, intravital microscopy in IRI models	(88, 89)
Metabolic adaptation during reperfusion	Support neutrophil metabolic reprogramming for controlled responses	Promote mitochondrial dysfunction, redox imbalance, and ferroptotic susceptibility in hepatocytes	mtROS, iron flux regulators, lipid peroxidation mediators	Ferroptosis inhibitors, iron chelation, mitochondrial probes	(90)
Immune signaling regulation	Modulate cytokine networks to balance early defense and repair	Amplify NF-κB and MAPK cascades; escalate pro-inflammatory cytokine release	DAMPs, PRRs, downstream inflammatory mediators	NF-κB/MAPK inhibition, cytokine profiling in HIRI	(91–93)
Spatiotemporal dynamics	Early low-level NET release supports rapid pathogen control and stabilization	Sustained or delayed NET accumulation collapses immune homeostasis, propagates pyroptosis and ferroptosis	NET–pyroptosis–ferroptosis regulatory components	Time-resolved imaging, reperfusion-time stratification, spatiotemporal NET mapping	(94, 95)

### 3.2 NETs-mediated pathological cascades: membrane damage and immune homeostasis collapse

NETs directly damage hepatocytes through multiple mechanisms. These NET-driven molecular events translate into clinically relevant injury endpoints by coupling membrane permeabilization and mitochondrial dysfunction to hepatocellular enzyme release (e.g., ALT/AST), while simultaneously promoting platelet–vWF–dependent microthrombosis and PRR-mediated cytokine maturation that amplify sterile inflammation (96). First, the strong positive charge of H3cit electrostatically binds to the negative charges of phospholipids in the hepatocyte membrane, forming transmembrane nanopores that increase membrane permeability (97, 98). This perforation effect not only triggers potassium ion efflux but also induces calcium ion influx, activating the calpain system and further disrupting the stability of cytoskeletal proteins, such as α-actinin and neuromuscular proteins (99, 100).

In the programmed necrosis cascade, the RIPK1/RIPK3 complex forms transmembrane pores through the phosphorylation of MLKL, synergistically amplifying the membrane perforation induced by NETs (101, 102). Studies have shown that the S100A8/A9 protein complex carried by NETs binds to the receptor for advanced glycation end products (RAGE), activating the ASK1-JNK/p38 signaling axis, promoting ROS bursts, and exacerbating membrane lipid peroxidation (103, 104). Additionally, MPO in NETs oxidizes mitochondrial inner membrane phospholipids, disrupting the electron transport chain complex IV and leading to mitochondrial membrane potential collapse (105). Mitochondrial damage further releases cytochrome C, activating the caspase-9-mediated apoptosis pathway and forming a synergistic necrosis-apoptosis effect (106). Notably, NE cleaves the Bcl-2 family protein MCL-1, releasing its inhibition on BAX/BAK and amplifying the opening of the mitochondrial outer membrane permeability transition pore (44, 107–109).

While directly damaging hepatocytes, NETs also disrupt microcirculation through a dual physical-biochemical mechanism. DNA components electrostatically bind to platelet membrane phospholipid serine (PS), increasing hepatic sinusoidal blood flow resistance. Simultaneously, ultra-high molecular weight von Willebrand factor (vWF) multimers released by endothelial cells promote platelet adhesion via the GPIbα receptor and upregulate markers, such as CD45/CD66b, on the surface of neutrophils, creating a “platelet-neutrophil-endothelial cell” vicious cycle (110, 111). Cathepsin G in NETs activates complement C5, generating C5a, which enhances neutrophil NADPH oxidase activity through the C5aR1 receptor, establishing a prothrombotic-proinflammatory feedback loop (112, 113). This microcirculatory dysfunction exacerbates ischemic injury and upregulates hepatic glucose transporter GLUT1 via hypoxia-inducible factor 1α (HIF-1α), further aggravating lactic acid accumulation and intracellular acidosis caused by glycolysis (114, 115).

NETs also activate the innate immune response via pattern recognition receptors (PRRs). The TLR9 pathway recognizes methylated CpG motifs, forming the MyD88-IRAK4 complex, which promotes the maturation of IL-1β/IL-18 precursors. Simultaneously, mtDNA induces type I interferon secretion via the cGAS-STING pathway (116, 117). The spatiotemporal coordination of these pathways is achieved through epigenetic regulation. TLR9-activated NF-κB enhances H3K4me3 modification in the STING promoter region, while STAT1 phosphorylation induced by the STING pathway further enhances TLR9 signaling, resulting in irreversible immunopathological damage (118, 119).

Additionally, HMGB1 enriched in NETs binds to the CXCL12 receptor to activate CXCR4, inducing HSCs to transform into myofibroblasts and promoting collagen deposition via the TGF-β/Smad3 pathway (120, 121). This multidimensional injury mechanism ultimately leads to the collapse of liver microenvironment homeostasis, providing a pathological basis for the progression of liver fibrosis.

### 3.3 Spatiotemporal dynamic regulation of NETs functions

The spatiotemporal regulatory mechanisms of NETs demonstrate their precise coordination between pathological and reparative functions. From a temporal perspective, the biological effects of NETs evolve in a stage-dependent manner as injury progresses. In the early ischemic phase, a small number of NETs recognize necrotic cell fragments via the phagocytic receptor CLEC5A, initiating clearance processes. The potentially destructive enzymes within NETs are inhibited by α1-antitrypsin, thus exerting a protective clearance role (122, 123). At this stage, mtDNA within NETs induces type I interferon secretion through the cGAS-STING pathway, activating the early expression of tissue repair-related genes without triggering an inflammatory storm (124, 125).

In the early phase of reperfusion, a surge of ROS triggers the translocation of the PAD4 enzyme into the cell nucleus, a process regulated by the SUMO protease SENP3. SENP3 removes SUMO modifications from PAD4, enhancing its binding capacity to chromatin, which accelerates histone H3 citrullination and drives the massive release of NETs (126, 127). The dense NETs formed at this stage activate the Syk kinase signaling axis through DNA-platelet glycoprotein VI (GPVI) interactions, promoting thromboxane A2 synthesis and significantly increasing microvascular blood flow resistance (107, 128). In the late phase of IRI, residual NETs bind to integrin αvβ6 receptors on hepatic stellate cells, activating the TGF-β/Smad signaling pathway and promoting collagen synthesis to stabilize the injured area (129, 130). This temporal regulation allows NETs to transition from an early protective role to a dual role in late-stage repair and fibrosis risk.

In the spatial dimension, the pathological effects of NETs exhibit significant heterogeneity depending on the anatomical microenvironment (131). In the central venous area with high blood flow shear stress, NETs-DNA binds to platelet surface GPIIb/IIIa receptors via electrostatic interactions, forming mechanically stable thrombus complexes that exacerbate local circulatory barriers (132, 133). Simultaneously, the hypoxic environment in this region upregulates CD73 expression on neutrophils via HIF-1α, promoting adenosine production that suppresses anticoagulant endothelial cell function, thereby forming a local prothrombotic vicious cycle (78, 134).

In the periportal zone, NETs activate the ERK1/2 signaling pathway in cholangiocytes by enriching epidermal growth factor (EGF), driving the expansion of the cholangiolar branching network and promoting bile duct regeneration (135, 136). This spatial difference is driven not only by physical factors but also by microenvironmental interactions of specific molecules. Neutrophils in the central venous zone perceive changes in vWF conformation through the SLC44A2 receptor, triggering NADPH oxidase-dependent ROS bursts (137). Meanwhile, NETs in the portal zone bind to fibronectin via α4β1 integrin, forming a three-dimensional scaffold that directs the extension of regenerating vessels (138).Metabolic reprogramming studies show that succinate released by portal zone NETs induces enhanced glycolysis in bile duct cells via the SUCNR1-GPR91 signaling axis, providing energy support for bile duct regeneration (139, 140).

**Figure 2:**
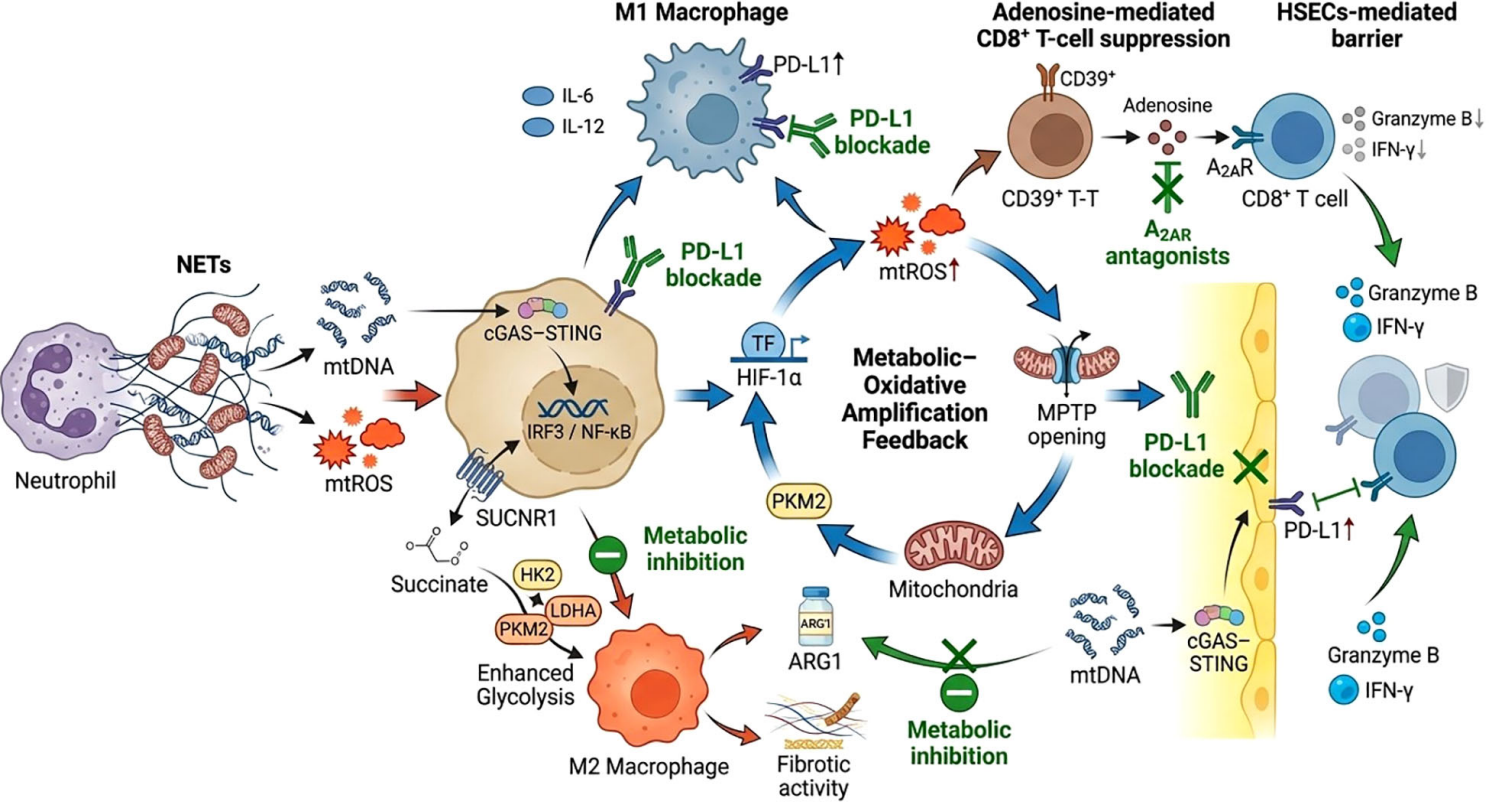
Integrated network linking NET-derived signals to macrophage polarization, metabolic amplification, immune suppression, and therapeutic targets. NETs release mitochondrial DNA (mtDNA) and mitochondrial ROS (mtROS), initiating inflammatory signaling. Extracellular mtDNA activates the cGAS–STING–IRF3/NF-κB pathway in macrophages, driving M1 polarization with increased IL-6, IL-12, and PD-L1 expression. Succinate–SUCNR1 signaling and enhanced glycolysis (HK2, LDHA, PKM2) promote M2 polarization and ARG1-dependent fibrotic activity. A central metabolic–oxidative loop, governed by the HIF-1α/PKM2 axis and MPTP opening, amplifies mtROS production. On the immune-suppressive axis, CD39^+^ double-negative T cells generate adenosine that activates A2AR on CD8^+^ T cells, reducing granzyme B and IFN-γ. Liver sinusoidal endothelial cells (HSECs) upregulate PD-L1 via cGAS–STING, forming a combined physical and biochemical barrier to cytotoxic T-cell activity. Therapeutic interventions—PD-L1 blockade, A2AR antagonists, and metabolic inhibitors targeting succinate or HK2—partially reverse NET-driven T-cell exhaustion by disrupting key immune–metabolic nodes.

The spatiotemporal dynamic regulation of NETs also achieves cross-dimensional coordination through epigenetic mechanisms. NETs-induced overexpression of DNA methyltransferase 3A (DNMT3A) in the central venous zone enhances fibrosis signal sensitivity in the portal tract by methylating the TGF-βR2 promoter region (141). Simultaneously, IL-33 secreted by bile duct cells in the portal tract reversely regulates the Notch signaling pathway in neutrophils in the central venous zone via the ST2 receptor, forming a bidirectional feedback regulatory network (142). This dynamic equilibrium system of “local injury amplification-targeted repair activation” (143) enables NETs to act not only directly on liver parenchymal cells but also to determine the final outcome of injury and repair by reshaping the immune microenvironment (**Figure 2**).

## 4 NETs and immune microenvironment interactions

### 4.1 Neutrophil heterogeneity drives spatiotemporal specificity of immune responses

**Figure 3:**
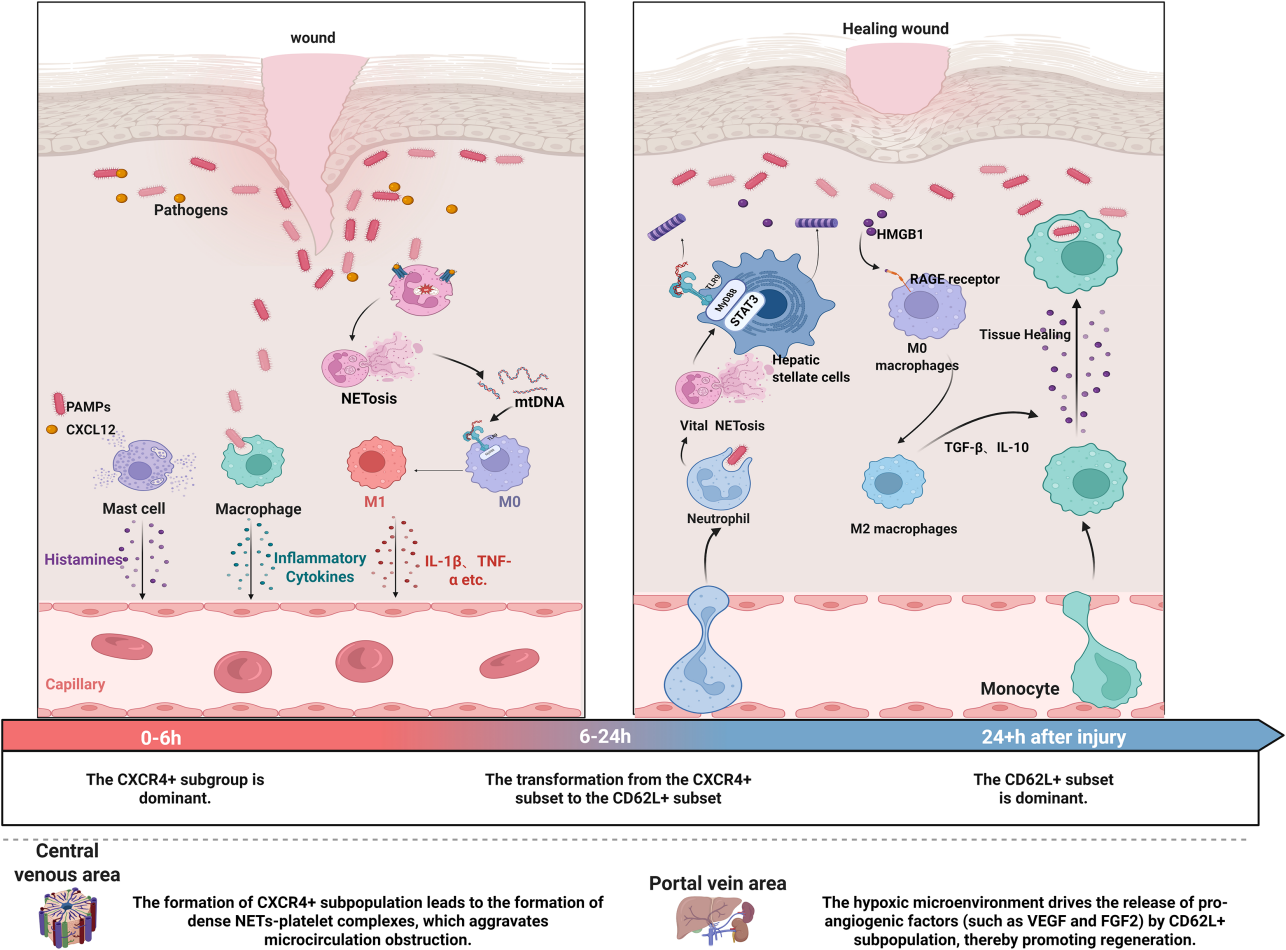
Temporal and functional heterogeneity of neutrophils during ischemia–reperfusion. During early ischemia (0–6 h), CXCR4^+^ neutrophils dominate, producing ROS via NADPH oxidase to drive NETosis and trap pathogens while TLR9–MyD88 signaling polarizes M1 macrophages, boosting inflammation through IL-1β and TNF-α. In reperfusion (6–24 h), CD62L^+^ neutrophils take over, releasing mtDNA‐rich NETs without killing cells; these activate TLR9/STAT3 in hepatic stellate cells to promote collagen deposition, angiogenesis, and M2 macrophage polarization, which secretes TGF-β and IL-10 for fibrosis repair. High shear in central venous zones fosters NET–platelet clots that worsen microcirculatory block, whereas portal‐vein hypoxia drives CD62L^+^ neutrophils to release VEGF and FGF2, enhancing tissue regeneration.

In HIRI, neutrophil subpopulation differentiation exhibits spatiotemporal functional plasticity, with the balance between pro-inflammatory (CXCR4+) and reparative (CD62L+) subpopulations determining the dual pathological and reparative effects of NETs (144). During the early ischemic phase, the CXCR4+ subpopulation induces NETosis via NADPH oxidase-dependent ROS bursts, releasing dense DNA networks to trap pathogens. This process also activates the TLR9-MyD88 pathway, which induces M1-type macrophage polarization (secreting IL-1β and TNF-α) ^[23]^, thereby amplifying the inflammatory response. In the later stages of reperfusion, the CD62L+ subpopulation releases non-lethal NETs via mtDNA, whose unmethylated CpG motifs activate the TLR9/STAT3 pathway in HSCs, promoting collagen deposition and angiogenesis. Additionally, this pathway induces M2-type macrophage transformation (secreting TGF-β and IL-10) (145, 146) via the HMGB1-RAGE axis, facilitating fibrotic repair. This spatiotemporal heterogeneity is regulated by microenvironmental parameters: within 6 hours post-ischemia, the CXCR4+ subset predominates, while the CD62L+ subset increases after 24 hours. The high shear stress environment in the central venous zone promotes the formation of dense NET-platelet complexes by the CXCR4+ subset, exacerbating microcirculatory obstruction. Meanwhile, the hypoxic microenvironment in the perivascular zone drives the CD62L+ subpopulation to release pro-angiogenic factors (e.g., VEGF and FGF2), promoting regeneration (**Figure 3**). This mechanism supports the theoretical framework that neutrophil heterogeneity synergistically regulates immune responses through three parameters—spatial, temporal, and disease context—providing a molecular basis for targeting the spatiotemporal-specific release of NETs (147, 148).

### 4.2 NETs regulate immune cell interaction networks

NETs dynamically regulate the phenotype and function of macrophages and T cells through multidimensional signaling pathways, forming a complex immune suppression network. At the macrophage level, NETs drive polarization and metabolic reprogramming via a dual signaling axis. On one hand, NET-DNA activates the cGAS-STING pathway, triggering type I interferon responses that induce M1-type macrophages to secrete pro-inflammatory factors (e.g., IL-6 and IL-12) and upregulate PD-L1 expression, suppressing the cytotoxic function of CD8+ T cells. On the other hand, succinate released by NETs enhances macrophage glycolysis (upregulating HK2 and LDHA expression) via the SUCNR1-GPR91 signaling axis, driving M2-type conversion and arginase 1 (ARG1) expression, which promotes tissue fibrosis repair (149, 150). Metabolic interactions further amplify pathological effects: mitochondrial ROS enhance glycolysis via the HIF-1α/PKM2 axis, forming a positive feedback loop with NET-induced mitochondrial membrane pore (MPTP) opening, exacerbating oxidative stress and promoting inflammatory factor release (151, 152). At the T cell level, NETs suppress anti-inflammatory functions through a metabolic-epigenetic dual mechanism. CD39+ double-negative T regulatory (DNT) cells activated by NET-DNA inhibit granzyme B and IFN-γ secretion in CD8+ T cells via the adenosine-A2AR signaling pathway. Additionally, NET-derived mtDNA induces PD-L1 expression in hepatic sinusoidal endothelial cells through the cGAS-STING pathway, creating a physical-biochemical barrier that restricts T cell infiltration (151, 153). Clinical data indicate that PD-L1 inhibitors can partially reverse NET-mediated T cell exhaustion (154), suggesting that targeting immune checkpoints may attenuate the immune escape effects of NETs. This multi-layered regulatory mechanism underscores the central role of NETs in reshaping the immune microenvironment, providing a theoretical foundation for developing time-specific intervention strategies.

### 4.3 NETs-mediated immune-metabolic interactions

NETs regulate iron metabolism and lactate signaling to remodel the immune microenvironment, forming a multi-layered metabolic-immune interaction network. In the context of ferroptosis, NETs-MPO depletes glutathione (GSH), leading to GPX4 inactivation. This, in turn, upregulates lipid peroxidation through ACSL4/LPCAT3, triggering macrophage ferroptosis and the release of DAMP molecules, such as HMGB1, which activate the NLRP3 inflammasome. Additionally, iron overload generates hydroxyl radicals via the Fenton reaction, causing mitochondrial membrane potential collapse in CD8+ T cells and inhibiting TCR signal transduction (as evidenced by downregulation of p-ZAP70), forming a vicious cycle of “NETs-iron overload-immune suppression” (6, 155). On the other hand, NET formation is closely associated with lactate metabolism. Pathogen metabolites, such as lactate produced by *Staphylococcus aureus*, enter neutrophil mitochondria via monocarboxylate transporters (MCT1), are converted to pyruvate by lactate dehydrogenase (LDH), and consume NAD+, inducing reverse electron transport (RET) and mitochondrial ROS bursts, which drive NETosis (156, 157). This metabolic reprogramming activates HIF-1α, which upregulates PAD4 expression, promoting histone H3 citrullination and the formation of NETs-DNA scaffolds (158, 159). Patients with systemic lupus erythematosus (SLE) exhibit abnormal NETosis due to mitochondrial lactate sensing defects, characterized by impaired anti-infective capacity and concurrent autoimmune overactivation (160, 161). This metabolic interaction not only amplifies the pathological effects of NETs but also inhibits T cell infiltration by inducing PD-L1 expression in hepatic sinusoidal endothelial cells. Clinical data suggest that PD-L1 inhibitors can reverse NETs-mediated T cell exhaustion, revealing the synergistic role of metabolic reprogramming and immune checkpoint regulation (131, 162).

### 4.4 NETs-mediated cross-organ immune regulation

NETs form a systemic immune regulatory network through cross-organ interactions between the gut-liver axis and the tumor microenvironment (163, 164). In the gut-liver axis, gut microbiota dysbiosis triggers dual pathological mechanisms via the portal vein circulation. Lipopolysaccharide (LPS) released by Gram-negative bacteria activates TLR4 on Kupffer cells in the liver, promoting CXCL1 secretion and neutrophil recruitment (129, 130). When the gut microbiota is imbalanced, the inversion of the Bacteroidetes/Firmicutes ratio significantly increases the release of gut-derived outer membrane vesicles (OMVs). These nanoparticles carry TLR9 ligands that directly induce ROS production in hepatic sinusoidal endothelial cells, synergistically enhancing the formation of NETs (165), thereby creating a vicious cycle: “microbiota dysbiosis → OMV transport → NET release → liver damage.” Additionally, MPO released from NETs can disrupt intestinal epithelial tight junctions, exacerbating microbiota translocation (166).

NETs also inhibit bile acid efflux pumps (BSEP) via the FXR signaling pathway, leading to the accumulation of toxic bile acids (e.g., deoxycholic acid [DCA]), which activate the TGR5 pathway in hepatic stellate cells, promoting fibrosis. Furthermore, the imbalance in secondary bile acid metabolism exacerbates intestinal barrier dysfunction (167), and DCA can activate the NLRP3 inflammasome to promote IL-1β secretion. This pro-inflammatory microenvironment accelerates liver fibrosis progression and induces neutrophil NETosis, forming a positive feedback loop (168). Imbalances in secondary bile acid metabolism suppress farnesoid X receptor (FXR) signaling, reducing FGF15/19 synthesis in the ileum and impairing intestinal barrier repair capacity (169). Recent studies have shown that abnormal accumulation of lithocholic acid (LCA) directly damages mitochondrial function in intestinal epithelial cells, inducing apoptosis through the BAX/BAK pathway, forming a “toxic bile acid-intestinal barrier disruption” secondary effect (170).

In the tumor microenvironment, NETs promote liver cancer recurrence through the chemokine-immunosuppression axis. The CXCL8/CXCR2 axis recruits myeloid-derived suppressor cells (MDSCs), which deplete arginine in the microenvironment via arginase-1 (Arg1), inhibiting CD8+ T cell proliferation (171). NETs-encapsulated histone H3cit can directly bind to Toll-like receptor 2 (TLR2) on T cell surfaces, upregulating PD-1 expression and reducing IFN-γ secretion, thereby promoting an acquired immune resistance phenotype (172, 173). Simultaneously, NETs-DNA-integrated VEGF and MMP-9 induce vascular endothelial cells to form tubular structures, promoting tumor vascular mimicry and blood supply reconstruction (174). Experimental evidence shows that NETs- NE cleaves E-cadherin, promoting epithelial-mesenchymal transition (EMT) and enhancing liver cancer cell stemness through activation of the PI3K/Akt pathway (175).

This cross-organ regulatory network underscores the pivotal role of NETs in inter-organ immune-metabolic interactions, providing a basis for combined interventions targeting gut-liver axis signaling and tumor microenvironment immune suppression pathways (**Table 3**). Imbalance in the immune microenvironment further amplifies pathological damage through the programmed cell death pathway, creating a cascade effect: “NETs-immune interaction-cell death” (176).

**Table 3:** NET interactions with immune and metabolic components in the hepatic microenvironment.

Hepatic component	Mode of interaction with NETs	Consequences for local immunity or metabolism	Key molecular mediators	Representative evidence/models	References
Kupffer cells	Detect NET-derived DAMPs and activate inflammatory signaling	Amplified cytokine release, enhanced phagocytic priming, propagation of sterile inflammation	HMGB1, DNA–protein complexes, inflammasome pathways	Kupffer cell depletion, DAMP neutralization, *in situ* imaging in IRI	(177)
Liver sinusoidal endothelial cells	Direct contact with NET structures and histones	Endothelial barrier disruption, increased permeability, sinusoidal congestion	Extracellular histones, proteases, ROS	Endothelial injury scoring, intravital microscopy	(178)
Hepatocytes	Uptake or exposure to NET-derived cytotoxic components	Membrane damage, mitochondrial stress, ferroptotic and necrotic susceptibility	mtROS, lipid peroxidation mediators, iron regulators	Ferroptosis inhibition, metabolic flux assays	(179)
Dendritic cells	NET-associated antigens regulate maturation state and antigen presentation	Skewed T-cell priming, heightened adaptive inflammatory tone	NET-bound nucleoproteins, PRR cascades	DC maturation assays, adoptive transfer experiments	(180)
Macrophage subsets	NETs modulate macrophage polarization dynamics	Shift toward pro-inflammatory phenotype; impaired resolution and repair	Cytokine networks, DNA-sensor signaling	Polarization markers, single-cell profiling	(181)
Platelets	Adhesion to NET structures and activation of coagulation pathways	Platelet aggregation, microthrombus formation, sinusoidal obstruction	Platelet adhesion molecules, vWF, histones	Platelet depletion, coagulation assays	(182)
T cells	NET components influence activation thresholds and cytokine patterns	Increased pro-inflammatory T-cell responses; reduced regulatory balance	NET-derived antigens, inflammatory mediators	T-cell activation assays, flow cytometry	(183)
Metabolic regulators	NETs perturb redox balance and metabolic signaling	Mitochondrial dysfunction, iron overload responses, enhanced lipid peroxidation	mtROS, iron-binding proteins, lipid oxidation enzymes	Metabolic profiling, redox sensor imaging	(184)
Stromal and non-parenchymal cells	Respond to NET-triggered cytokine and chemokine gradients	Altered microenvironmental homeostasis, impaired regeneration niches	Chemokines, matrix-interacting factors	Spatial transcriptomics, microenvironment mapping	(185)

## 5 Interactions between NETs and programmed cell death

NETs not only directly mediate inflammatory responses in HIRI but also modulate pathological processes by regulating programmed cell death, including pyroptosis and ferroptosis (106). Recent studies have shown that NETs interact with hepatic cell pyroptosis and ferroptosis through the release of specific molecules and oxidative stress products, offering new insights into the molecular pathophysiology of HIRI (186).

### 5.1 NETs activate the hepatic cell pyroptosis pathway via the HMGB1-TLR4 signaling axis

HMGB1 released by NETs acts as a DAMP by binding to TLR4 receptors on hepatocytes, thereby activating the NLRP3 inflammasome complex (187). In this axis, HMGB1 functions as the upstream DAMP, TLR4/NF-κB provides the priming signal for NLRP3 and pro–IL-1β, and caspase-1–mediated GSDMD cleavage constitutes the execution step that produces IL-1β/IL-18 release as the downstream inflammatory readout.TLR4 signaling triggers the NF-κB pathway, upregulating the expression of NLRP3, pro-caspase-1, and pro-IL-1β (188). This process is regulated by mitochondrial fusion protein 2 (MFN2); MFN2 deficiency exacerbates mitochondrial ROS release, thereby amplifying inflammasome activation (189). Concurrently, mtDNA derived from NETs enhances inflammasome assembly via the STING pathway (190), where STING activation not only promotes IRF3-dependent type I interferon production but also enhances NLRP3 oligomerization through TBK1 phosphorylation. Activated caspase-1 cleaves GSDMD, and its N-terminal fragment forms pores in the cell membrane, leading to the release of IL-1β/IL-18 and cellular swelling, ultimately triggering pyroptosis (170). ATP released during pyroptosis acts as a secondary DAMP, activating NADPH oxidase in neighboring neutrophils via P2X7 receptors, thereby promoting NET formation. Studies have shown that inhibiting the HMGB1-TLR4 axis significantly reduces hepatic cell pyroptosis and improves liver function in HIRI mouse models (182, 190). Notably, the pyroptosis cascade further promotes NET formation: released IL-1β recruits more neutrophils to the injury site and upregulates PAD4 expression via the IL-1R/MyD88 signaling pathway, inducing histone citrullination. This “NETs-pyroptosis-inflammatory amplification” feedback loop is supported by clinical correlative evidence—serum HMGB1 levels in liver transplant patients at 6 hours post-surgery were significantly correlated with NET marker MPO-DNA complex concentrations. Recent mechanistic studies have shown that ROS released from pyroptotic hepatocytes can activate the neutrophil PKCδ/ERK pathway, inducing NET formation and the release of new mtDNA (191).

### 5.2 NETs drive ferroptosis cascades through iron metabolism dysregulation

NET-released ROS synergistically drive hepatic iron death through a dual pathological mechanism. First, iron metabolism dysregulation is a key component: ROS convert Fe²^+^ to Fe³^+^ through the Fenton reaction while inhibiting the expression of the iron transport protein FPN, leading to the accumulation of intracellular free iron. This process is regulated by mitochondrial ferritin autophagy. NETs-released NE cleaves nuclear receptor coactivator 4 (NCOA4), accelerating ferritin autophagy body formation and releasing large amounts of Fe²^+^ into the unstable iron pool. Iron overload further catalyzes lipid peroxidation, generating toxic lipid free radicals (e.g., MDA and 4-HNE), which damage cellular membrane integrity (192). Additionally, iron overload promotes the transformation of HSCs into myofibroblasts by activating the Hippo/YAP pathway.

Second, the imbalance in the antioxidant system exacerbates damage: MPO in NETs consumes GSH, inhibits GPX4 activity, and weakens the cell’s ability to clear lipid peroxidation products (189). NETs-derived H3cit can directly bind to the catalytic subunit of glutathione S-transferase (GST), inhibiting the activity of the rate-limiting enzyme in GSH biosynthesis, thereby reducing GSH synthesis efficiency (193). GPX4 inactivation leads to the accumulation of phospholipid hydroperoxides (PLOOH), ultimately triggering ferroptosis (190).

NETs drive a vicious cycle of hepatic ferroptosis through multiple mechanisms, with the upregulation of ACSL4 and LPCAT3 expression as core components. ROS released by NETs catalyze iron overload via the Fenton reaction, activating the HIF-1α and Nrf2-Keap1 axes to promote the transcription of ACSL4 and LPCAT3. ACSL4 catalyzes the conversion of polyunsaturated fatty acids into easily oxidizable acyl coenzyme A, while LPCAT3 esterifies them into phospholipids, significantly increasing cellular membrane oxidative sensitivity. MtDNA fragments released by NETs can activate the ATM-Chk2 pathway, inducing dysfunction of iron-sulfur proteins (e.g., FDX1), further amplifying the Fenton reaction effect (194). Concurrently, NETs induce METTL3-dependent m6A modification via the cGAS-STING pathway, enhancing HIF-1α mRNA stability and further upregulating the expression of lipid metabolic enzymes (195).

Lipid peroxidation products (such as 4-HNE and MDA) form a positive feedback loop by activating the NF-κB and AP-1 pathways, while GPX4 activity decreases due to glutathione depletion, ultimately leading to an irreversible cycle of “iron overload → lipid peroxidation → upregulation of metabolic enzymes → exacerbated ferroptosis” (196). Based on the aforementioned mechanisms, recent animal experiments have confirmed the efficacy of a dual intervention strategy: targeting the clearance of NETs to block the source release of ROS and MPO, while iron death inhibitors (such as Ferrostatin-1) directly inhibit downstream lipid peroxidation processes. The synergistic effects of these two approaches significantly reduce hepatic cell damage. When combined with DNase I and the iron chelator deferoxamine (DFO), these interventions resulted in a significant reduction in liver tissue MDA levels and restoration of serum ALT/AST ratios to normal ranges. Additionally, novel strategies activating mitochondrial autophagy show promise: uric acid A restores Fundc1-mediated mitochondrial quality control, significantly restoring mitochondrial membrane potential in hepatocytes and markedly reducing the expression of ferroptosis-related proteins ACSL4 and COX2.

### 5.3 Spatiotemporal dynamic regulation of NETs synergistically exacerbates liver injury through pyroptosis and ferroptosis

The interaction between NETs and programmed cell death exhibits spatiotemporal dynamics. In the early stages of injury, NETs predominantly drive pyroptosis through the HMGB1-TLR4 axis, with the mechanism involving variations in the redox state of HMGB1. Reduced HMGB1 preferentially activates the TLR4/NLRP3 inflammasome, whereas oxidized HMGB1 enhances mitochondrial reactive oxygen species (ROS) release through the RAGE receptor, triggering the pyroptosis cascade (165). During this phase, mitochondrial DNA released by NETs promotes type I interferon secretion via the cGAS-STING pathway, which synergistically enhances inflammasome activation efficiency. ATP released through GSDMD pores during pyroptosis acts as a secondary damage-associated molecular pattern, activating inflammatory signals in neighboring neutrophils and forming a self-reinforcing inflammatory amplification loop.

As injury progresses, ROS derived from NETs drive the ferroptosis process by disrupting iron metabolism and collapsing the antioxidant system (197). Mitochondrial dysfunction enhances ferritin autophagy, lysosomal iron release activates the Fenton reaction, and elastase-mediated inhibition of glutathione synthesis further weakens cellular antioxidant capacity (198). At this stage, epigenetic regulatory mechanisms stabilize the transcriptional activity of hypoxia-inducible factors, upregulate the expression of lipid metabolism enzymes, and significantly enhance cellular membrane sensitivity to oxidative stress (176).

**Figure 4:**
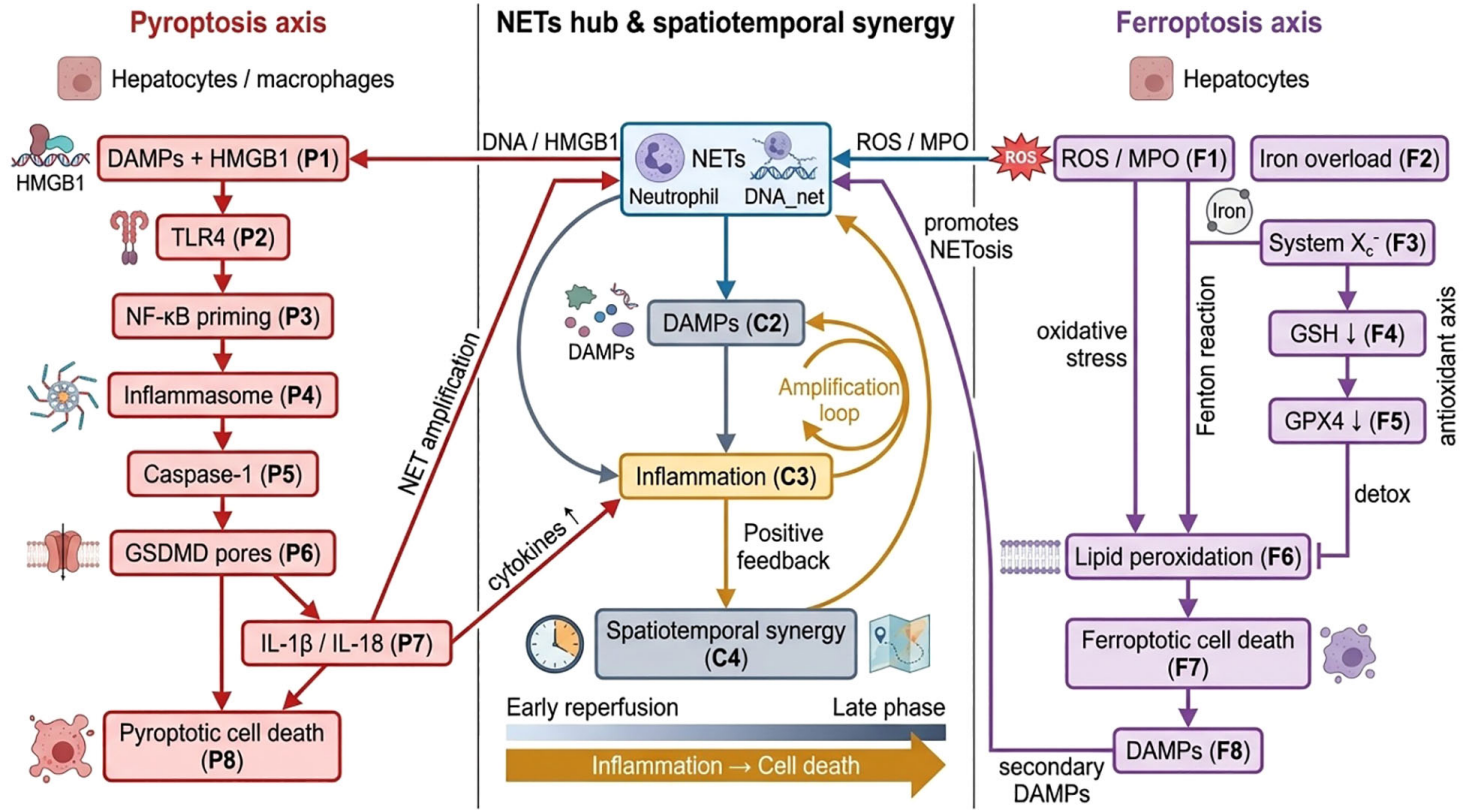
NETs couple pyroptosis and ferroptosis to amplify liver IRI. NETs orchestrate a synergistic amplification of liver ischemia–reperfusion injury by coupling pyroptosis and ferroptosis into interconnected death circuits. On the pyroptotic axis, NET-associated DAMPs, including extracellular DNA and histones, together with HMGB1–TLR4 engagement, provide inflammatory priming and inflammasome activation. This cascade promotes caspase-1 activation, gasdermin D (GSDMD) pore formation, and the release of IL-1β and IL-18, which intensify local inflammation and further stimulate NET formation, establishing a self-reinforcing inflammatory loop. In parallel, the ferroptotic axis is driven by NET-induced oxidative stress and iron dysregulation. NET-associated ROS and MPO activity exacerbate lipid peroxidation in the setting of increased labile iron, while impairment of the System Xc^-^/GPX4 antioxidant axis weakens cellular redox defense. The resulting accumulation of lipid hydroperoxides triggers ferroptotic cell death, accompanied by secondary DAMP release that feeds back into NETosis and inflammatory escalation. A central integrative hub highlights the spatiotemporal synergy between these pathways. During early reperfusion, inflammatory amplification predominates, favoring NET-driven cytokine release and immune activation. As injury progresses, sustained oxidative stress and metabolic imbalance shift the response toward cell-death–dominated re-amplification, whereby pyroptosis and ferroptosis mutually reinforce tissue damage. Together, this coordinated network transforms NETs from passive inflammatory byproducts into active determinants of injury severity, positioning the NET–pyroptosis–ferroptosis axis as a critical target for stage-specific therapeutic intervention in liver ischemia–reperfusion injury.

This temporal regulation suggests that targeting intervention points at different stages may be more effective. Early blockade of HMGB1 signaling can suppress the inflammatory cascade triggered by pyroptosis, while combining iron metabolism regulation and antioxidant therapy in later stages can effectively alleviate the progression of ferroptosis (199). Experimental evidence indicates that spatiotemporally precise intervention strategies—synergistically targeting NETs clearance and mitochondrial quality control—can significantly reduce hepatic cell damage and restore tissue homeostasis, providing new insights into balancing the pathological damage and repair functions of NETs (175) (**Figure 4**).

## 6 Advances in therapeutic target research: from traditional strategies to precision medicine

### 6.1 Traditional intervention strategies: mechanism exploration and clinical translation bottlenecks

**Figure 5:**
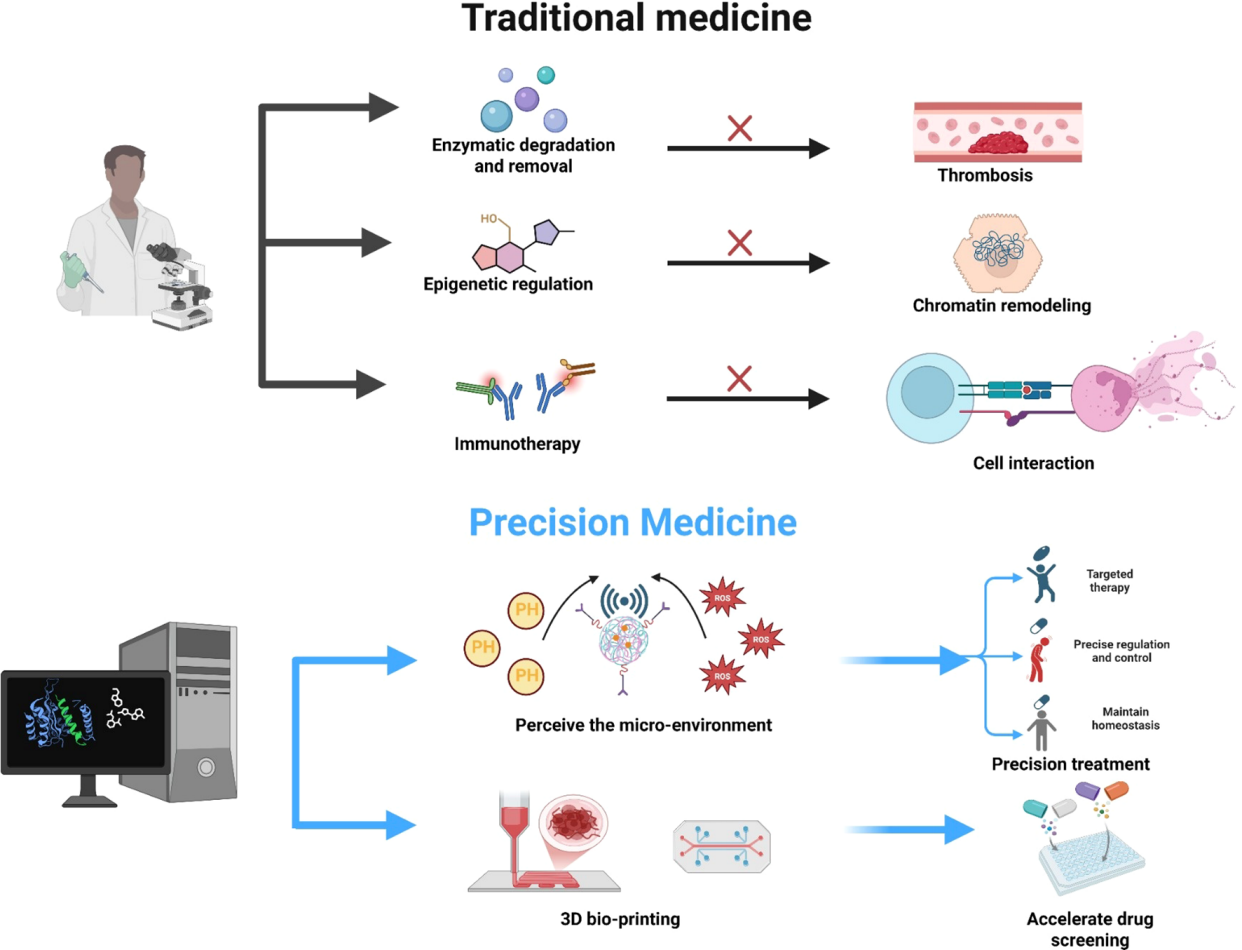
Conventional versus precision strategies for targeting NETs and inflammatory pathways. Traditional medicine involves broad strategies like enzymatic degradation, epigenetic regulation, and immunotherapy, but faces limitations in treating complex conditions like thrombosis, chromatin remodeling, and cell interactions. Precision medicine, on the other hand, focuses on individualized approaches. It includes micro-environment perception, 3D bio-printing, and targeted therapy, offering precise regulation and homeostasis maintenance. Precision medicine also accelerates drug screening, enabling more effective and personalized treatments.

Traditional intervention strategies focus on directly blocking the pathogenic pathways of NETs; however, their clinical application is limited by the conflicting demands of efficacy and safety (200). At the mechanistic level, existing strategies primarily target three approaches: enzymatic clearance through the degradation of NET DNA backbones using engineered DNases, combined with elastase inhibitors to mitigate their prothrombotic effects (174); epigenetic regulation targeting PAD4-mediated histone citrullination using small-molecule inhibitors or natural compounds (such as baicalein) to intervene in chromatin remodeling (**Figure 5**) (201); and immune blockade therapy, which targets abnormal interactions between NETs and immune cells through neutralizing antibodies or complement inhibitors (163). These strategies have demonstrated potential in reducing liver injury markers (e.g., ALT, AST) in animal models, such as PD-1/PD-L1 inhibitors significantly alleviating liver injury. However, the clinical translation of traditional strategies encounters two major bottlenecks. First, the narrow therapeutic window poses challenges, as NETs exhibit pro-inflammatory effects in the early stage of injury (<6 hours) and reparative functions in the later stage (>24 hours), making it difficult for systemic administration to achieve precise temporal control. Second, widespread inhibition of NETs may disrupt their physiological defense functions. Clinical data indicate that DNase therapy increases the risk of postoperative infection, while PAD4 inhibitors induce hepatotoxicity at high doses (187). These limitations highlight the inadequacy of single-target interventions and underscore the urgent need for new strategies that integrate spatiotemporal specificity and functional selectivity.

### 6.2 New directions in precision medicine: spatiotemporal dynamic regulation and interdisciplinary integration

Wang et al. highlighted that the emergence of spatiotemporal molecular medicine signifies the entry of clinical and translational medicine into a new era. Precision medicine is overcoming the spatiotemporal limitations of traditional treatment strategies through multidimensional technological innovations, forming a closed-loop system of “smart delivery-dynamic decision-making-simulation validation.” Qu et al. (116) demonstrated that smart drug delivery systems utilize novel nanomaterials (such as magnetic nanocarriers) to sense the microenvironmental characteristics of lesions (such as reactive oxygen species concentration and pH changes), achieving spatiotemporal-specific drug release. For example, nanocarriers encapsulating DNases can accumulate in damaged liver areas under magnetic field guidance and precisely intervene in oxidative stress responses by degrading the DNA backbone of NETs; CRISPR-Cas9 carriers coated with neutrophil membranes can target inflammatory genes for knockout by leveraging cell homing properties, thereby avoiding systemic side effects (170). Meanwhile, Yan et al. (79) found that 3D-bioprinted miniature liver chips can mimic the ischemia-reperfusion microenvironment, reproducing hepatic sinusoidal structures and immune interactions in a centimeter-scale device, thereby accelerating drug screening processes. For example, this platform identified that low-dose baicalin administered six hours before reperfusion selectively inhibits pathological NETs, with related findings advancing to phase II clinical trials (183). These technologies, through spatiotemporal dynamic regulation and multidisciplinary integration, have collectively achieved a paradigm shift from crude treatment to precise intervention (**Table 4**).

**Table 4:** Therapeutic strategies targeting NETs — from traditional interventions to precision approaches.

Strategy/agent	Mechanism of action	Model evidence	Advantages	Limitations and safety considerations	References
DNase I	Degrades extracellular DNA scaffold of NETs, promoting NET disassembly	Preclinical HIRI and sepsis models; clinical use in other indications	Rapid structural NET disruption; immediate reduction of NET burden	Short half-life; potential off-target effects on extracellular DNA-dependent host functions; dosing and local delivery challenges	(202)
PAD4 inhibitors	Inhibit histone citrullination, preventing chromatin decondensation required for NET formation	Multiple rodent models of NET-associated injury	Targeted blockade of a central NET formation step	Potential effects on physiological citrullination processes; chronic inhibition safety unknown	(168)
Neutrophil elastase inhibitors	Block proteolytic histone cleavage and nuclear envelope disruption	Preclinical HIRI and inflammatory models	Direct inhibition of a NET-promoting effector; oral and parenteral agents available	Risk of impaired antimicrobial defense; off-target protease effects	(203)
MPO inhibitors	Reduce oxidative contributions to chromatin decondensation and NET stability	Preclinical inflammation models	Attenuates oxidative NET-mediated tissue injury	Possible impairment of host microbial killing; long-term safety unestablished	(182)
Anti-DAMP/PRR blockade	Neutralize damage signals or block pattern recognition receptor signaling to reduce NET priming	Preclinical HIRI and sterile inflammation studies	Interrupts upstream amplification of NETosis and inflammation	Broad immunomodulation risk; specificity depends on target selection	(185)
Platelet–neutrophil interaction inhibitors	Disrupt adhesion and signaling that prime vascular NET formation	Intravital imaging and IRI rodent models	Reduces localized NET deposition and microthrombosis	May increase bleeding risk; effects on hemostasis require careful titration	(204)
Antioxidants/NOX2 modulators	Reduce ROS generation that drives classical NETosis	Preclinical HIRI and oxidative stress models	Dampens a central NET-triggering axis; potential broad cytoprotective effects	ROS have physiological roles; systemic suppression may impair host defense and signaling	(180)
Iron chelators and ferroptosis inhibitors	Limit iron-catalyzed lipid peroxidation that couples NETs to ferroptotic pathways	HIRI models integrating NET and ferroptosis readouts	Targets NET–metabolic crosstalk; may protect hepatocytes from lipid peroxidation	Systemic iron modulation can cause anemia; off-target metabolic effects	(178)
NET-targeted nanodelivery	Deliver NET-disrupting agents or gene modulators specifically to neutrophils or NET-rich microenvironments	Proof-of-concept in rodent models of inflammatory injury	Enhanced local concentration, reduced systemic exposure, potential for controlled release	Complexity of formulation, scale-up, biodistribution, immunogenicity concerns	(188)
siRNA/gene editing targeting NET regulators	Silence or edit genes required for NET formation in neutrophils	Preclinical cell and animal studies	High specificity; potential for durable modulation	Delivery barriers to neutrophils; ethical and safety concerns for gene editing	(205)
Immune-modulatory biologics	Modulate downstream inflammatory amplification triggered by NETs	Clinical and preclinical studies in related inflammatory disorders	Leverages established clinical agents; can reduce cascade effects of NETs	May not reduce NET structures directly; infection risk with immune suppression	(206)
Combined spatiotemporal precision strategies	Time-restricted and site-directed therapy integrating NET disruption with organ-protective measures	Emerging preclinical paradigms incorporating reperfusion timing	Balances NET removal with preservation of host defense; aligns with pathophysiologic time windows	Requires accurate biomarkers and delivery platforms; operational complexity for clinical deployment	(192)

## 7 Conclusions and outlook

NETs exhibit complex bimodal regulatory characteristics in HIRI, with molecular heterogeneity and spatiotemporal specificity playing pivotal roles in determining the balance between injury and repair. Research suggests that NETs dynamically regulate oxidative stress, programmed cell death, and the inflammatory microenvironment, establishing a sequential pathological network marked by an “early pro-inflammatory phase followed by a late pro-fibrotic phase.” While traditional intervention strategies target key enzymes involved in NET formation or aim to clear DNA scaffolds, they are constrained by narrow therapeutic windows and insufficient functional selectivity. Recent studies indicate that the integration of multi-dimensional technologies, such as genomics and proteomics, provides deeper insights into the mechanisms linking NETs to organelle damage. This research is also driving the development of advanced nanodrugs capable of sensing the microenvironment at the lesion site, thereby enabling precise, spatiotemporal control over NET release patterns. These mechanistic insights also provide a practical roadmap for translation. Stage-resolved NET signatures can be leveraged to distinguish NOX2-dependent, ROS-driven and Cit-H3–enriched NETosis from mtDNA-enriched extracellular traps, and then integrated with routine graft-injury markers to build time-stratified risk models and define optimal sampling windows for early diagnosis and prognosis. Therapeutically, mechanism-linked stratification supports matching interventions to the dominant NET program—such as early redox/priming control, mid-phase inhibition of chromatin remodeling, or localized NET dismantling—thereby maximizing efficacy while limiting impairment of antimicrobial defense. Future research should focus on integrating multi-level data, including the use of single-cell technology to analyze the spatiotemporal interactions between NET subtypes and ferroptosis, and combining imagingomics with metabolomics to create clinical prediction models. This closed-loop system of “mechanism elucidation—smart intervention—personalized prediction” will advance the prevention and treatment of post-liver transplantation injury from empirical approaches to a new era of precision medicine.

## Glossary

HIRI: hepatic ischemia–reperfusion injury
IRI: ischemia–reperfusion injury
NETs: neutrophil extracellular traps
NETosis: neutrophil extracellular trap formation
ROS: reactive oxygen species
RNS: reactive nitrogen species
mtROS: mitochondrial reactive oxygen species
mtDNA: mitochondrial DNA
NOX2: NADPH oxidase 2
PAD4: peptidyl arginine deiminase 4
H3cit: citrullinated histone H3
MPO: myeloperoxidase
NE: neutrophil elastase
PKC: protein kinase C
MAPK: mitogen-activated protein kinase
NF-κB: nuclear factor kappa B
HMGB1: high-mobility group box 1
RAGE: receptor for advanced glycation end products
TLR: Toll-like receptor
TLR4: Toll-like receptor 4
TLR9: Toll-like receptor 9
MyD88: myeloid differentiation primary response 88
cGAS: cyclic GMP–AMP synthase
STING: stimulator of interferon genes
IRF3: interferon regulatory factor 3
IFN: interferon
PRRs: pattern recognition receptors
Ca²⁺: calcium ion
ER: endoplasmic reticulum
ATG: autophagy-related protein
MPTP: mitochondrial permeability transition pore
HIF-1α: hypoxia-inducible factor 1 alpha
JMJD3: Jumonji domain-containing protein 3
JNK: c-Jun N-terminal kinase
PFKFB3: 6-phosphofructo-2-kinase/fructose-2,6-bisphosphatase 3
CXCR4: C-X-C chemokine receptor 4
CD62L: L-selectin
HSCs: hepatic stellate cells
HSECs: hepatic sinusoidal endothelial cells
VEGF: vascular endothelial growth factor
FGF2: fibroblast growth factor 2
MMP-9: matrix metalloproteinase 9
ANGPT2: angiopoietin 2
FAK: focal adhesion kinase
Src: Src family kinase
COL1A1: collagen type I alpha 1 chain
M1: classically activated macrophage
M2: alternatively activated macrophage
IL: interleukin
TNF-α: tumor necrosis factor alpha
TGF-β: transforming growth factor beta
STAT3: signal transducer and activator of transcription 3
PD-1: programmed cell death protein 1
PD-L1: programmed death-ligand 1
T cells: cluster of differentiation 8 positive T cells
DNT cells: double-negative T cells
CD39: ectonucleoside triphosphate diphosphohydrolase 1
A2AR: adenosine A2A receptor
SUCNR1 (GPR91): succinate receptor 1
HK2: hexokinase 2
LDHA: lactate dehydrogenase A
PKM2: pyruvate kinase M2
ARG1: arginase 1
GSH: glutathione
GPX4: glutathione peroxidase 4
ACSL4: acyl-CoA synthetase long-chain family member 4
LPCAT3: lysophosphatidylcholine acyltransferase 3
MDA: malondialdehyde
4-HNE: 4-hydroxynonenal
GSDMD: gasdermin D
NLRP3: NOD-like receptor family pyrin domain-containing 3
Caspase-1: cysteine-aspartic protease-1
RIPK1: receptor-interacting protein kinase 1
RIPK3: receptor-interacting protein kinase 3
MLKL: mixed lineage kinase domain-like protein
vWF: von Willebrand factor
GPVI: glycoprotein VI
GPIbα: glycoprotein Ib alpha
GPIIb/IIIa: glycoprotein IIb/IIIa
FXR: farnesoid X receptor
BSEP: bile salt export pump
DCA: deoxycholic acid
LCA: lithocholic acid
TGR5: G protein-coupled bile acid receptor 1
FGF15/19: fibroblast growth factor 15/19
MCT1: monocarboxylate transporter 1
LDH: lactate dehydrogenase
RET: reverse electron transport
OMVs: outer membrane vesicles
LPS: lipopolysaccharide
MDSCs: myeloid-derived suppressor cells
EMT: epithelial–mesenchymal transition
PI3K: phosphoinositide 3-kinase
Akt: protein kinase B
DNMT3A: DNA methyltransferase 3A
m6A: N6-methyladenosine
METTL3: methyltransferase-like 3
Keap1: Kelch-like ECH-associated protein 1
Nrf2: nuclear factor erythroid 2–related factor 2
HO-1: heme oxygenase 1
DNase I: deoxyribonuclease I
DFO: deferoxamine
ALT: alanine aminotransferase
AST: aspartate aminotransferase
SLE: systemic lupus erythematosus.
